# Supplementary material for: Association between community-based resource collection site use and functional disability risk among older adults: A Quasi-experimental study
Source: PLoS One. 2025 Oct 15;20(10):e0332327. doi: 10.1371/journal.pone.0332327 (PMC12527121; doi:10.1371/journal.pone.0332327)
Supplement: S1 Fig — (1) Ikoma City (Community A). (2) Tachiarai Town (Hongo community). (3) Tachiarai town (Ozeki community). (DOCX) [file pone.0332327.s001.docx]

# **Supporting Information**

**S1 Fig. Appearance of MEGURU STATION and its users.**

(1) Ikoma City (Community A)

(2) Tachiarai Town (Hongo community)

(3) Tachiarai town (Ozeki community)
