# Supplementary material for: Association between community-based resource collection site use and functional disability risk among older adults: A Quasi-experimental study
Source: PLoS One. 2025 Oct 15;20(10):e0332327. doi: 10.1371/journal.pone.0332327 (PMC12527121; doi:10.1371/journal.pone.0332327)
Supplement: S1 Table — (DOCX) [file pone.0332327.s002.docx]

# **Supporting Information**

**S1 Table. List of functions by MEGURU STATION installation area**

| **Ikoma (Community A)** | |
| --- | --- |
| **Functions** | **Description** |
| Vegetable direct sales | Sales of vegetables grown by local farmers |
| Volunteer | Volunteers to operate MEGURU STATION installation sites |
| Check-in card registration | Registration of ICT-based check-in systems |
| Use check-in system | Use the check-in system at the entrance of MEGURU STATION |
| Bringing kitchen waste | Bring resource trash generated at home |
| Free distribution of liquid fertilizer | Collection and utilization of liquid fertilizer discharged from resource-collection site |
| Greenway Cafe | Simple cafe set up by MEGURU STATION |
| Event | Events held in areas where MEGURU STATION is installed (e.g., Christmas parties, outing promotion events) |
| **Hongo** | |
| **Functions** | **Description** |
| AOZORA market | Sales and free donations (sharing) of vegetables grown by local farmers |
| MEGURU farm | Simple farm by volunteers |
| **Ozeki** | |
| **Functions** | **Description** |
| Cake sales | Cake sales by residents |
