# Supplementary material for: Association between community-based resource collection site use and functional disability risk among older adults: A Quasi-experimental study
Source: PLoS One. 2025 Oct 15;20(10):e0332327. doi: 10.1371/journal.pone.0332327 (PMC12527121; doi:10.1371/journal.pone.0332327)
Supplement: S2 Table — Note: Opportunities changed due to visits to MEGURU STATION installation sites in the past six months, from 1 year after installation. Data are presented as n (%). * Multiple answers were allowed. (DOCX) [file pone.0332327.s003.docx]

# **Supporting Information**

**S2 Table. Subjective changes due to MEGURU STATION use**

|  |  |  |  | MEGURU STATION | | p-value |
| --- | --- | --- | --- | --- | --- | --- |
|  |  | Total |  | Use | Non-use |  |
|  |  | N=973 |  | n=187 | n=786 |  |
| Opportunities to interact with others | Greatly increased | 21 (4.5) |  | 11 (6.1) | 10 (3.4) | < 0.001 |
|  | Increased | 143 (30.4) |  | 77 (43.0) | 66 (22.7) |  |
|  | Hardly increased | 156 (33.2) |  | 56 (31.3) | 100 (34.4) |  |
|  | Not increased | 150 (31.9) |  | 35 (19.6) | 115 (39.5) |  |
| Age groups of people you interacted with*, years | 12 or younger | 7 (1.6) |  | 4 (2.3) | 3 (1.1) | 0.520 |
|  | 13-18 | 2 (0.4) |  | 1 (0.6) | 1 (0.4) |  |
|  | 19-39 | 1 (0.2) |  | 0 (0.0) | 1 (0.4) |  |
|  | 40-64 | 17 (3.8) |  | 4 (2.3) | 13 (4.7) |  |
|  | 65-74 | 145 (32.4) |  | 61 (35.1) | 84 (30.7) |  |
|  | 75 or older | 276 (61.6) |  | 104 (59.8) | 172 (62.8) |  |
| Opportunities to participate in community activities | Greatly increased | 13 (2.7) |  | 9 (5.0) | 4 (1.4) | < 0.001 |
|  | Increased | 112 (23.5) |  | 61 (33.7) | 51 (17.2) |  |
|  | Hardly increased | 166 (34.8) |  | 64 (35.4) | 102 (34.5) |  |
|  | Not increased | 186 (39.0) |  | 47 (26.0) | 139 (47.0) |  |
| Opportunities to go out | Greatly increased | 13 (2.7) |  | 9 (5.0) | 4 (1.4) | < 0.001 |
|  | Increased | 160 (33.8) |  | 79 (43.9) | 81 (27.6) |  |
|  | Hardly increased | 180 (38.1) |  | 65 (36.1) | 115 (39.2) |  |
|  | Not increased | 120 (25.4) |  | 27 (15.0) | 93 (31.7) |  |

Note: Opportunities changed due to visits to MEGURU STATION installation sites in the past six months, from 1 year after installation.

Data are presented as n (%).

* Multiple answers were allowed.
