# Supplementary figures and images for: Association between community-based resource collection site use and functional disability risk among older adults: A Quasi-experimental study
Source: PLoS One. 2025 Oct 15;20(10):e0332327. doi: 10.1371/journal.pone.0332327 (PMC12527121; doi:10.1371/journal.pone.0332327)

# **Supporting Information**

**S2 Fig. Residuals vs. fitted values plot**


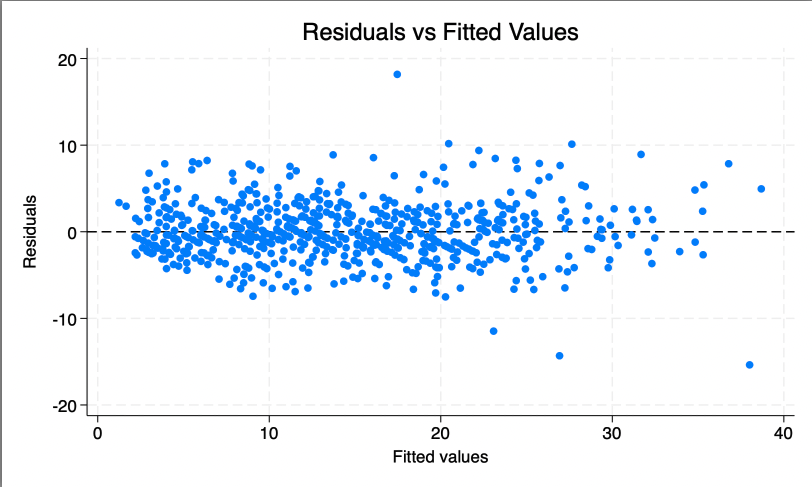

Supplement: S2 Fig — (DOCX) [file pone.0332327.s004.docx]
