# Supplementary material for: Association between community-based resource collection site use and functional disability risk among older adults: A Quasi-experimental study
Source: PLoS One. 2025 Oct 15;20(10):e0332327. doi: 10.1371/journal.pone.0332327 (PMC12527121; doi:10.1371/journal.pone.0332327)
Supplement: Attachment — Submitted filename: peer review.docx [file pone.0332327.s005.docx]

1. General Assessment

This manuscript presents a novel and well-executed quasi-experimental study that examines whether the use of MEGURU STATION—a community-based resource collection hub—can reduce the risk of functional disability among older adults in Japan. The topic is timely and addresses a growing need for sustainable, socially inclusive aging interventions that can be embedded within existing infrastructure. The study aligns with current global priorities on healthy aging and social participation.

2. Strengths

Originality:
The intervention concept is innovative—leveraging daily activities such as waste disposal to promote social participation. This represents a creative application of primordial prevention.

Methodological Rigor: The use of a quasi-experimental design with a clear intervention and comparison group, stratified analyses, multiple imputation for missing data, and sensitivity analyses (e.g., E-value) is commendable and enhances causal inference in the absence of randomization.

Data Presentation: Results are presented with clarity. The tables are informative, and the supplementary data provide transparency.

Relevance:
The findings have clear implications for public health policy, especially in rapidly aging societies like Japan. The intervention is potentially scalable and adaptable.

3. Points for Improvement

3.1. Clarification on Temporal Relationship

The study reports a follow-up of one year after baseline, but the exact timing of MEGURU STATION implementation in each community relative to data collection should be clearly stated to reinforce causal interpretation.

3.2. Generalizability

The authors acknowledge cultural limitations; however, they may further discuss how community-based interventions like MEGURU STATION could be tailored in non-Japanese or lower-resource settings.

3.3. Participant Selection and Bias

As the baseline response rate was 59.7% and follow-up was 68.8%, please discuss the potential impact of response bias on generalizability or internal validity.

3.4. Functional Disability Measure

While the RSFD is validated, it is still a predictive score rather than actual disability incidence. The limitations section appropriately acknowledges this, but the authors might briefly outline any plans or ongoing efforts to assess actual LTCI certification or disability onset in future work.

3.5. Sex and Age Stratified Analyses

In the stratified results (S2 Table), the benefits appear more pronounced in younger older adults and in women. Consider discussing possible gender-role or mobility differences that may influence these patterns.

4. Minor Revisions

Language: A few sentences can benefit from light grammatical refinement for flow, particularly in the introduction and discussion. However, overall English usage is standard and understandable.

Terminology: Consider defining RSFD in full the first time in both abstract and main text.

Ethical Compliance: Ethical approval and consent processes are clearly stated and conform to PLOS standards.

5. Recommendation

The study is of high relevance and technical quality, with findings that merit publication. The manuscript requires only minor clarification and slight elaboration in the discussion.
