# Supplementary material for: Association between community-based resource collection site use and functional disability risk among older adults: A Quasi-experimental study
Source: PLoS One. 2025 Oct 15;20(10):e0332327. doi: 10.1371/journal.pone.0332327 (PMC12527121; doi:10.1371/journal.pone.0332327)
Supplement: Attachment — Submitted filename: Response_to_Reviewers.docx [file pone.0332327.s006.docx]

**Response to Reviewers**

We sincerely thank both reviewers for their detailed and constructive feedback. Below, we have provided point-by-point responses to each comment. All changes have been incorporated into the revised manuscript, with the tracked changes visible for clarity.

**Response to Reviewer #1**

Thank you for your thoughtful and encouraging feedback. Below are our point-by-point responses to your suggestions:

Comment 1: The baseline and follow-up of one year require more exact timing of hub implementation using more detailed data collection.

Response 1: Thank you for pointing this out. We have now clarified the timing of MEGURU STATION implementation relative to baseline and follow-up data collection for each community. Specifically, we added the installation dates to the revised: in Ikoma (Community A), installation was completed in December 2020, which was shortly after the baseline survey in November 2020; in the Hongo community, the baseline survey was conducted in February 2022 and the installation occurred in April 2022; and in the Ozeki community, both occurred in September 2022. The follow-up surveys were conducted approximately one year after installation in all communities. This information has been added to the end of the "Study design" subsection of the Methods section.

(see P6, L122-126: *MEGURU STATION was installed in December 2020 in Community A, April 2022 in the Hongo community, and September 2022 in the Ozeki community. Follow-up surveys were conducted approximately 1 year after installation: November 2021 in Community A, July 2023 in the Hongo community, and December 2023 in the Ozeki community.*)

Comment 2: The perspective of using this Japanese community-based intervention for non-Japanese or lower-resource settings.

Response 2: In this Discussion section, we have elaborated on how MEGURU STATION may be adapted to non-Japanese or resource-constrained settings by emphasizing the concept of integrating social participation into daily community activities. Specifically, we have clarified that similar models could be explored in resource-constrained environments by incorporating social interaction into familiar community spaces (e.g., markets or shared public areas), without requiring major infrastructure changes.

(see P24, L402–407: *Although MEGURU STATION was developed in a Japanese cultural context, its core concept—integrating opportunities for social participation into routine community activities—may be adaptable to various cultural and resource settings. In resource-constrained environments, similar models could be explored by incorporating social interaction into familiar community spaces, such as community markets or shared public areas, without requiring major infrastructure changes.*)

Comment 3: The relatively low response rate and follow-up rate could also be interpreted.

Response 3: We appreciate the reviewer’s comment. Accordingly, we have revised the Limitations section by clarifying the potential impact of response bias as a distinct limitation. Specifically, we have added a separate statement discussing the possible distortion of findings due to differences between respondents and non-respondents, such as greater social isolation or poorer health among non-respondents.

(See P23, L396–P24, L401: *Finally, the baseline and follow-up response rates were 59.7 % and 68.8 %, respectively. Although these are typical for mail-based surveys, response bias may have affected the representativeness of the findings. Specifically, if non-respondents systematically differed from respondents—for example, if non-respondents were more socially isolated or had poorer health—the observed associations between MEGURU STATION use and RSFD could be underestimated.*)

Comment 4: Could the predictive tool be extended or updated for early functional disability screening, which could be mirrored by a more pronounced tendency in younger older adults and in women.

Response 4: We sincerely appreciate this valuable suggestion. The RSFD is a validated screening tool that is designed to predict long-term care certification, and our study focused on applying this established tool. While we did not pursue the development of an updated version in this study, we recognize the potential importance of refining or expanding such tools, particularly to reflect early-stage disability risks among younger older adults and women. We hope that future research will explore this direction.

Comment 5: PS. English grammar could be refined.

Response 5: Thank you for your comment. We have carefully revised the manuscript for grammar and clarity and have had the final version reviewed by a native English speaker with academic writing experience.

**Response to Reviewer #2**

We sincerely thank Reviewer #2 for their thorough and constructive comments, which have significantly helped us improve the clarity and rigor of our manuscript. We are grateful for the positive recognition of our study’s strengths, including its real-world relevance, use of mixed-effects models, and sensitivity analyses. We have carefully reviewed each of the points raised and have revised the manuscript accordingly. Below, we provide detailed point-by-point responses to each comment and indicate where specific revisions have been made.

Comment 1: Causal Inference & Confounding

As a non-randomized study, residual confounding remains a concern. Although the E-value (CI limit = 2.01) suggests only strong unmeasured confounders could negate the association, baseline differences (users were older, more socially active, and had lower initial RSFD; Table 1) warrant further adjustment or discussion. Propensity-score adjustment or matching could strengthen causal claims.

Response 1: Thank you for this important comment. We fully acknowledge the risk of residual confounding in observational studies such as ours. To mitigate this, our primary analyses adjusted for baseline RSFD along with 15 additional covariates (age, sex, ADL, social participation, subjective economic status, etc.; see the “Covariates” section for details).

Because our data come from three distinct regions—each with locally tailored MEGURU STATION implementations—we employed mixed-effects linear models with random intercepts for region. This approach allowed us to simultaneously account for individual-level confounders and regional heterogeneity, which we believe is the most appropriate strategy given the hierarchical structure of our data.

We recognize that propensity-score methods (e.g., matching or weighting) could offer complementary control of observed confounding. We have therefore added a note to the Limitations section acknowledging that future work should explore such methods to further address potential biases.

(see P22, L365–P23, L380: *This study has several limitations. First, residual confounding remains a concern owing to the non-randomized design of this quasi-experimental study. To mitigate this, we adjusted for baseline RSFD and 15 covariates, including demographic, socioeconomic, and health-related variables, using a mixed-effects linear regression model. This approach was chosen specifically to account for clustering by community, reflecting the fact that MEGURU STATION features varied by district policy and context. Additionally, the potential impact of unmeasured confounding was assessed using the E-value (CI limit = 2.01). Among covariates included in the model, only age and participation in neighborhood associations exceeded this threshold. Given that MEGURU STATION is often located near venues where neighborhood association meetings are held, the influence of unmeasured confounders beyond these factors is likely limited. While propensity-score methods (e.g., matching or weighting) were considered as an alternative strategy to further balance observed covariates, we did not apply them in this study due to concerns that our relatively small MEGURU STATION user sample (n = 187) could lead to unstable estimates and extreme weights. Future research with larger sample sizes should implement propensity-score approaches as an additional sensitivity analysis to strengthen causal inference.*)

Comment 2: Causal Inference & Confounding

Clarify whether baseline RSFD was included as a covariate in the regression (methods mention adjustment for RSFD*). If not, adding baseline RSFD would control for regression to the mean.

Response 2: Thank you for your comment. We confirm that baseline RSFD was included as a covariate in our regression models to adjust for individual differences in initial risk levels and to control for regression to the mean. This is explicitly stated in the “Covariates” section of the Methods. In response to the reviewer’s comment, we have also added a description in the manuscript clarifying that baseline RSFD was included to consider initial risk differences and to control for regression to the mean.

(see P9, L189–190: *The RSFD was included as a covariate to control for participants' initial differences in functional status and to account for potential regression to the mean.*)

Comment 3: Clinical Relevance of Effect Size

A coefficient of –1.20 on a 0–48 scale is statistically significant, but its practical impact on preventing long-term care certification is unclear. Please contextualize this change: e.g., what shift in absolute risk or number needed to treat does this represent?

Response 3: Thank you for this insightful comment. As noted, the RSFD is a continuous predictive measure rather than a diagnostic threshold or categorical outcome, making it inherently challenging to calculate an exact number needed to treat (NNT). However, previous validation work (Tsuji et al., 2018) has shown that each 1-point increase in RSFD is associated with a 1.128-fold higher risk of new-onset functional disability over three years. The observed reduction of 1.20 points among MEGURU STATION users suggests a possible contribution to reducing future long-term care certification risk.

Comment 4: Measurement of Exposure

MEGURU STATION “use” is self-reported at follow-up. Provide details on the survey question wording, frequency thresholds for classification as “user,” and any validation of self-report against logs or observations.

Response 4: We appreciate the reviewer’s thoughtful comment regarding the measurement of MEGURU STATION use. As noted, “use” was based on participants’ self-reported responses in the follow-up survey. While we did not available usage logs or observational data for validation—owing to privacy concerns and the absence of consent for using such records in this study—we took several steps to ensure classification accuracy.

Participants were asked to select from a list of activities available at the community center, which included both MEGURU STATION-related functions and other unrelated programs. Only those who selected one or more predefined activities officially identified by municipalities as MEGURU STATION functions (e.g., resource collection, vegetable sales, greenway relaxation) were classified as “users.” The classification was based on municipal documentation and in consultation with local officials during survey design. Furthermore, MEGURU STATION was actively promoted through local newsletters and community announcements, and its facilities were familiar to residents owing to their daily proximity to these communication challenges. Therefore, while self-report bias cannot be entirely ruled out, we believe that participants could accurately recognize and report their use of MEGURU STATION.

(see P7, L152–P8, L166: *The classification of MEGURU STATION users was based on self-reported data from the follow-up survey. Participants were asked to select all activities they had engaged in at the community center, from a list including both MEGURU STATION-related functions (e.g., resource collection, vegetable sales, café use, greenway relaxation) and unrelated programs (e.g., community salons, hobby clubs). Only those who selected at least one predefined activity officially designated as part of the MEGURU STATION program by municipal authorities were classified as users. These activities were identified in advance through consultation with the respective local governments and documentation of MEGURU STATION operations.*

*While usage logs or observational data were not available owing to privacy concerns and the absence of consent for research use, MEGURU STATION facilities were widely promoted through local newsletters and community announcements and were familiar landmarks in participants’ daily routines. Therefore, although the possibility of self-report bias cannot be entirely excluded, we believe that participants could reliably recognize and report their use of MEGURU STATION.*)

Comment 5: Data Availability

PLOS ONE requires that all data underlying findings be fully available. “Participants needed to provide consent for their data to be publicly shared” is insufficient. The authors should deposit de-identified data in a public repository or explain ethical/legal restrictions and provide a route for qualified researchers to access data.

Response 5: Thank you for pointing this out. We have revised the Data Availability Statement to clarify the ethical and legal restrictions. The dataset is owned by local insurers and was obtained under joint research agreements. Owing to these agreements and municipal personal information protection policies, the data cannot be shared publicly. However, qualified researchers may request access to the anonymized dataset by contacting Chiba University. We have also stated that we were unable to obtain permission to publish the questionnaire owing to local administrative restrictions. These revisions have been reflected in both the manuscript and submission form.

(see P25, Data availability:

*The dataset used in this study cannot be publicly shared owing to ethical and legal restrictions. Although participant consent was obtained for research use, it did not include permission for public data sharing. Furthermore, data are owned by local insurers and were obtained under joint research agreements. Data access is restricted by the personal information protection committees of the respective municipalities.*

*Qualified researchers may request access to the anonymized dataset by contacting the Research Ethics Committee of Chiba University (email: inohana-rinri@chiba-u.jp).*

*Additionally, the questionnaire used in this study was developed based on the JAGES framework (https://www.jages.net/). However, we were unable to obtain permission from the relevant municipality to publish it, either as Supporting Information or via an online repository.*)

Comment 6: Statistical Methods

Provide justification for choosing linear regression on RSFD rather than a generalized linear model if RSFD is skewed or bounded. Include diagnostic checks (e.g., residual plots).

Response 6: We appreciate the reviewer’s thoughtful comment regarding the appropriateness of the linear regression model for RSFD. As suggested, we have conducted a residual analysis to assess the assumptions of linear regression. The residual vs. fitted values plot showed that residuals were approximately symmetrically distributed around zero and did not exhibit clear patterns or trends, indicating that the linearity assumption was reasonably met. Although slight heteroskedasticity was observed, it was not substantial enough to warrant the use of alternative modeling approaches. These findings support the validity of using a linear regression model. We have included the residual plot as S2 Fig for reference.

**S2 Fig. Residuals vs. fitted values plot.**

Comment 7: Statistical Methods

Describe handling of missing data in covariates: percent missing, patterns, and convergence diagnostics for imputation.

Response 7: Thank you for pointing this out. We have now described the proportion of missing data in the manuscript. Specifically, missing values ranged from 1.8% (sex) to 11.8% (RSFD at follow-up). The RSFD score is a composite indicator; therefore, the proportion of missing values was slightly higher owing to multiple item requirements. Excluding RSFD, the variable with the highest missing rate was participation in sports groups (4.6%). We performed multiple imputations under the assumption of missing at random (MAR), using the multivariate normal regression method. Twenty imputed datasets were generated and combined using Rubin’s rule. The imputation model included all variables used in the analysis.

(see P10, L202–206: *The missing values for the covariates ranged from 1.8 % (sex) to 11.8 % (RSFD at follow-up). Because the RSFD is a composite index based on multiple items, a relatively higher rate of missingness was observed. Excluding RSFD, the highest missing rate among covariates was 4.6 % for participation in sports groups.*)

Comment 8: Ethics & Funding Statements

The funding statement lists two sources but lacks grant numbers associated with specific authors and funder URLs. Please format per PLOS guidelines.

Response 8: Thank you for pointing this out. Accordingly, we have revised the Funding section to comply with PLOS ONE’s policy. The statement now includes the funders’ names, grant number (JPMJOP1831), and a clarification that the funders had no role in study design, data collection and analysis, decision to publish, or preparation of the manuscript.

Comment 9: Clarity and Style

In Table 1, clarify “Use of other MEGURU STATION functions*” – what counts as “other” versus core functions?

Response 9: We appreciate the reviewer’s comment. Accordingly, we have clarified the meaning of “other MEGURU STATION functions” in Table 1. Specifically, “core functions” refer to waste collection activities, while “other functions” refer to peripheral community activities such as cafés, vegetable stands, and community events. We have added a footnote to Table 1 to explain this distinction.

(see P7, L143–144 and Table 1)

Comment 10: Clarity and Style

Replace “Coef.” with “B” (unstandardized beta) for consistency with Stata output.

Response 10: Thank you for pointing this out. Accordingly, we have revised all occurrences of “Coef.” to “B.”

Comment 11: Clarity and Style

Ensure uniform formatting of numbers (e.g., space before “%” in tables).

Response 11: Thank you for your comment. Accordingly, we have carefully revised the manuscript and all tables to ensure consistent number formatting. Specifically, we inserted a space before the percentage symbol (%) in accordance with standard formatting conventions. We also reviewed all numeric values to maintain consistent presentation throughout the manuscript and tables.

Comment 12: Introduction & References

Reference [19] (Amita HD marketing material) is a non–peer-reviewed source. Consider citing a peer-reviewed description or non-commercial report.

Check that all URLs in references are active and include access dates.

Response 12: Thank you for your comment. We acknowledge that Reference [19] is not a peer-reviewed source. However, to the best of our knowledge, no peer-reviewed or non-commercial academic publications describing MEGURU STATION exist at this time. Because this study is the first academic investigation of MEGURU STATION, we have retained the current reference to provide necessary contextual background, despite its non-peer-reviewed nature. We have confirmed that all URLs cited in the references are currently active and have updated the reference list to include access dates for each web-based source.

Comment 13: Figures & Supporting Information

Figure 1 (flowchart) is clear, but axes and labels (e.g., reasons for exclusion) could be enlarged for readability. S1–S4 Tables are informative; consider moving S2 and S4 into the main text to highlight stratified and mechanism analyses.

Response 13: We appreciate your thoughtful feedback regarding Figure 1 and Supplementary Tables.

To improve readability, we have enlarged the font size of all labels in Figure 1, including exclusion criteria, and have adjusted spacing to enhance clarity.

In addition, to emphasize the stratified and mechanism analyses, we moved S2 Table and S4 Table into the main text as Table 4 and Table 5, respectively.

Comment 14: Discussion

The discussion of cognitive stimulation via waste sorting is intriguing but speculative. Tone this down or cite direct evidence linking sorting tasks to cognitive benefit.

Response 14: Thank you for this insightful comment. We have revised the discussion to acknowledge the speculative nature of this mechanism. Specifically, we now state that “direct evidence linking this activity to cognitive benefit is limited,” while retaining a plausible explanation grounded in the cognitive demands of sorting tasks. The revised paragraph maintains a cautious tone and clarifies that the potential benefit is hypothetical and supported indirectly by previous literature on cognitive stimulation.

(see P21, L342–343: *While sorting plastics, paper, cans, and bottles requires attention, memory, and executive function, direct evidence linking this activity to cognitive benefit is limited.*)
